# Did Large-Scale Vaccination Drive Changes in the Circulating Rotavirus Population in Belgium?

**DOI:** 10.1038/srep18585

**Published:** 2015-12-21

**Authors:** Virginia E. Pitzer, Joke Bilcke, Elisabeth Heylen, Forrest W. Crawford, Michael Callens, Frank De Smet, Marc Van Ranst, Mark Zeller, Jelle Matthijnssens

**Affiliations:** 1 Department of Epidemiology of Microbial Diseases, Yale School of Public Health, New Haven, Connecticut, United States of America; 2 Fogarty International Center, National Institutes of Health, Bethesda, Maryland, United States of America; 3 Centre for Health Economics Research & Modeling of Infectious Diseases (CHERMID), Vaccine and Infectious Disease Institute (VAXINFECTIO), University of Antwerp, Wilrijk, Antwerp, Belgium; 4 KU Leuven - University of Leuven, Department of Microbiology and Immunology, Laboratory for Clinical and Epidemiological virology, Rega Institute for Medical Research, Leuven, Belgium; 5 Department of Biostatistics, Yale School of Public Health, and Department of Ecology and Evolutionary Biology, Yale University, New Haven, Connecticut, United States of America; 6 National Alliance of Christian Sickness Funds, Brussels, Belgium; 7 KU Leuven – University of Leuven, Department of Public Health and Primary Care, Environment and Health, Leuven, Belgium

## Abstract

Vaccination can place selective pressures on viral populations, leading to changes in the distribution of strains as viruses evolve to escape immunity from the vaccine. Vaccine-driven strain replacement is a major concern after nationwide rotavirus vaccine introductions. However, the distribution of the predominant rotavirus genotypes varies from year to year in the absence of vaccination, making it difficult to determine what changes can be attributed to the vaccines. To gain insight in the underlying dynamics driving changes in the rotavirus population, we fitted a hierarchy of mathematical models to national and local genotype-specific hospitalization data from Belgium, where large-scale vaccination was introduced in 2006. We estimated that natural- and vaccine-derived immunity was strongest against completely homotypic strains and weakest against fully heterotypic strains, with an intermediate immunity amongst partially heterotypic strains. The predominance of G2P[4] infections in Belgium after vaccine introduction can be explained by a combination of natural genotype fluctuations and weaker natural and vaccine-induced immunity against infection with strains heterotypic to the vaccine, in the absence of significant variation in strain-specific vaccine effectiveness against disease. However, the incidence of rotavirus gastroenteritis is predicted to remain low despite vaccine-driven changes in the distribution of genotypes.

Rotavirus gastroenteritis (RVGE) is estimated to kill more than 450,000 children below the age of 5 years annually^1^. Since 2009 the WHO has recommended the inclusion of rotavirus vaccines in national immunization schedules worldwide^2^. Currently two live, oral rotavirus vaccines, Rotarix® (GlaxoSmithKline Biologicals, Rixensart, Belgium) and RotaTeq® (Merck and Co., Whitestation, NJ, USA), are licensed in most countries around the world^3^.

Group A Rotaviruses (RVAs) are double-stranded RNA viruses with a genome composed of 11 segments^4^,^5^. While a large diversity of RVA strains has been described in literature, only two major RVA genotype constellations (Wa-like and DS-1-like) are known to cause the vast majority of RVGE in humans. Despite the rather conserved nature of these human RVA genomes, the two outer capsid proteins, VP7 and VP4, are much more variable in comparison with the rest of the genome^6^. These two outer capsid proteins both elicit neutralizing antibodies, and are classified into distinct G- (VP7) and P- (VP4) genotypes^7^,^8^. In high-income countries, the DS-1-like human RVAs are usually found in combination with G2 and P[4], whereas the Wa-like human RVA strains are usually found in combination with G1, G3, G4, G9, G12 and P[8]^6^,^9^,^10^,^11^,^12^,^13^,^14^,^15^,^16^,^17^,^18^. Human RVAs with various G/P-genotype combinations are known to co-circulate in any given location at any given time, and strong fluctuations in their distributions are known to occur from year to year and from one location to another^19^. Currently, the reasons for these fluctuations are poorly understood, although homotypic and heterotypic immunity due to natural RVA exposure or vaccination are believed to play important roles, in addition to several other stochastic factors^20^.

Rotavirus vaccines, available since 2006, have been successful at controlling the burden of RVGE in countries with large-scale vaccination programs. The two available vaccines differ in their approach to eliciting immunity^21^. The Rotarix vaccine consists of an attenuated Wa-like G1P[8] RVA strain, whereas RotaTeq contains a cocktail of 5 bovine RVA strains (distinct from the typical human Wa- and DS-1-like RVA strains), where each strain contains either a human VP7 (G1, G2, G3 and G4) or VP4 (P[8]) gene, introduced by *in vitro* reassortment^22^. Although there is a broad scientific consensus that both vaccines are highly effective in reducing the burden of severe RVGE, there is still controversy regarding the long-term effects of vaccination on the circulating RVA genotype distribution^20^,^23^. Changes in the genotype distribution have been observed following large-scale vaccination with Rotarix and/or RotaTeq in the United States, some Australian states, and Brazil^20^,^24^,^25^,^26^,^27^, but it remains unclear if these changes can be attributed to vaccination. In Belgium, where primarily Rotarix is used, a clear increase in the proportion of RVGE cases caused by the G2P[4] genotype was noted after vaccine introduction, and has lasted now for at least 7 seasons (2006–2013)^28^,^29^ [unpublished data]. In addition, a recent study found strong statistical differences in the genotype distribution of vaccinated and unvaccinated children, with an increased prevalence of G2P[4] in vaccinated children in Belgium, suggesting that Rotarix may exert selective pressures on the viral population^30^.

Mathematical models can provide insight into the underlying dynamics of rotavirus and changing rotavirus populations. However, ours is the only model thus far to have explicitly accounted for the interaction of multiple strains of rotavirus that co-circulate^31^. Moreover, very few rotavirus models have been fitted to post-vaccination data^32^. The lack of strain-specific rotavirus models may be due to uncertainties in the many parameters needed to describe the interaction of different strains and the difficulties associated with fitting complex epidemiological models to data. The one strain-specific model for rotavirus demonstrated that the cycling of genotypes in the population could be explained by differences in homotypic and heterotypic immunity, and predicted that vaccination with a monovalent vaccine such as Rotarix could exert different pressures on the viral population than a vaccine that provided strong protection again all strains, such as RotaTeq^31^. However, the model did not differentiate between Wa-like and DS-1-like rotavirus strains and the differences in immunity that may result.

Here, we utilize mathematical models to explore the hypothesis that selection pressures imposed by large-scale rotavirus vaccination can explain the observed impact of vaccination and changes to the genotype distribution in Belgium. We employ a hierarchy of models fit to multiple sources of data to estimate key parameters to refine model predictions for the long-term impact of vaccination and to explain the predominance of G2P[4] following vaccine introduction in Belgium.

## Results

### Impact of vaccination on the overall incidence of RVGE

#### Observed impact

**Figure 1:**
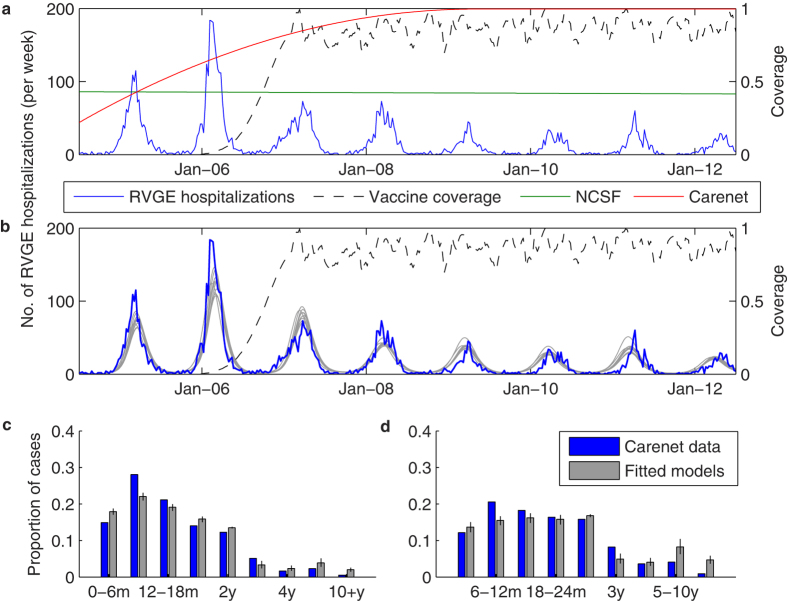
Rotavirus hospitalizations in Belgium from July 2004 to June 2012. (**a**) Number of weekly hospitalizations with a rotavirus diagnosis reported in the Carenet-NCSF database (blue) is plotted along with the proportion of the Belgian population who receive their health insurance through NCSF (green) and the proportion of hospital beds included in the Carenet database (red). The interpolated weekly vaccination coverage with at least one dose is indicated by the dashed black line. (**b**) The output of the non-strain-specific models fit to the Carenet-NCSF data is plotted in grey, while the number of weekly rotavirus gastroenteritis (RVGE) hospitalizations reported in Carenet-NCSF (blue) and vaccination coverage (dashed black line) are plotted as above. (**c**) The age distribution of rotavirus hospitalizations in Carenet-NCSF prior to vaccine introduction (July 2004-June 2006) is indicated by the blue bars, while the grey bars represent the mean of the fitted models. The black error bars represent the range of the fitted models. (**d**) The age distribution of rotavirus hospitalizations after vaccine introduction (July 2006-June 2012) for the Carenet-NCSF data (blue) and the fitted models (grey).

Rotavirus vaccination coverage with at least one dose increased from 0% to 75% during 2006, and the coverage with either vaccine has levelled off at approximately 86% (Fig. 1a,b, Fig. S1); however, only 79% of infants received a full course of either vaccine in 2012 (Fig. 1a, Fig. S1). Since the start of vaccination, the number of rotavirus related hospitalizations recorded by Carenet-NCSF has decreased by 85% (Fig. 1a). The largest decrease occurred in patients <2 years of age (67%, Fig. 1c,d). A more detailed description of the overall impact of vaccination on rotavirus burden in Belgium can be found in a separate paper^33^.

#### Predicted impact

**Figure 2:**
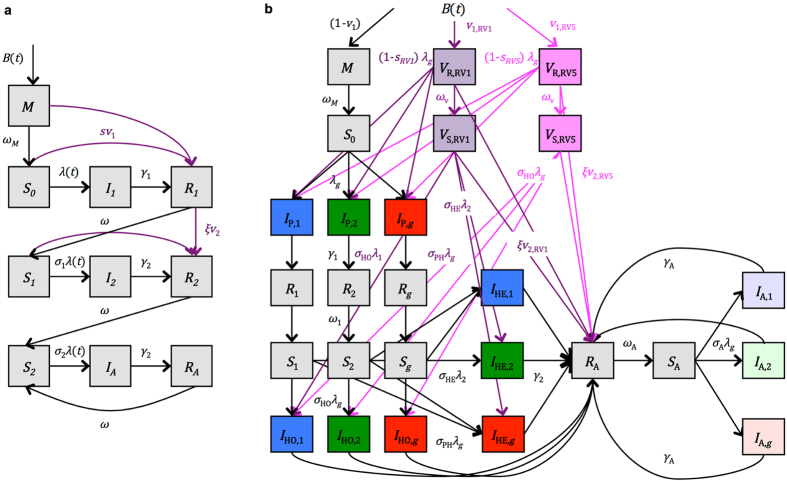
Compartmental diagrams illustrating model structures. (**a**) Non-strain-specific model. (**b**) Strain-specific model. Symbols are explained in the Methods section.

Our non-strain-specific model (Fig. 2a) was able to provide a good fit to the Carenet-NCSF data, although there was a tendency to slightly overestimate the peak number of rotavirus hospitalizations recorded during the 2006/07 season (Fig. 1b). The model was able to capture the age distribution of hospitalized cases well for children 1–4 years old, but slightly overestimated the proportion of hospitalizations in infants <6 months of age and underestimated the proportion of cases in 6–11 month olds both before and after vaccine introduction, and overestimated the proportion of cases among ≥5 year olds following vaccine introduction (Fig. 1c,d).

#### Parameter estimates

A variety of estimated parameter combinations were able to provide a good fit to the Carenet-NCSF data, both visually (Fig. 1) and statistically as indicated by comparing the log posterior probability (up to a normalizing constant) of the fitted models (Table 1). Estimates of the basic reproductive number, *R*_0_ (an important measure of transmissibility, defined as the expected number of secondary infections generated by one infectious individual in a fully susceptible population) varied from 13.6 to 21.6, and were negatively correlated with estimates of the relative infectiousness of asymptomatic infections (Pearson’s *r* = −0.98, *p* < 0.0001) (Table 1, Fig. S2). Most models estimated that ~50% of moderate-to-severe RVGE cases result in hospitalization with a rotavirus diagnosis among children <2 years old; reporting rates were estimated to be 69–85% lower among older children and adults, for whom the cost of rotavirus testing is not reimbursed (Table 1).

#### Model validation

**Figure 3:**
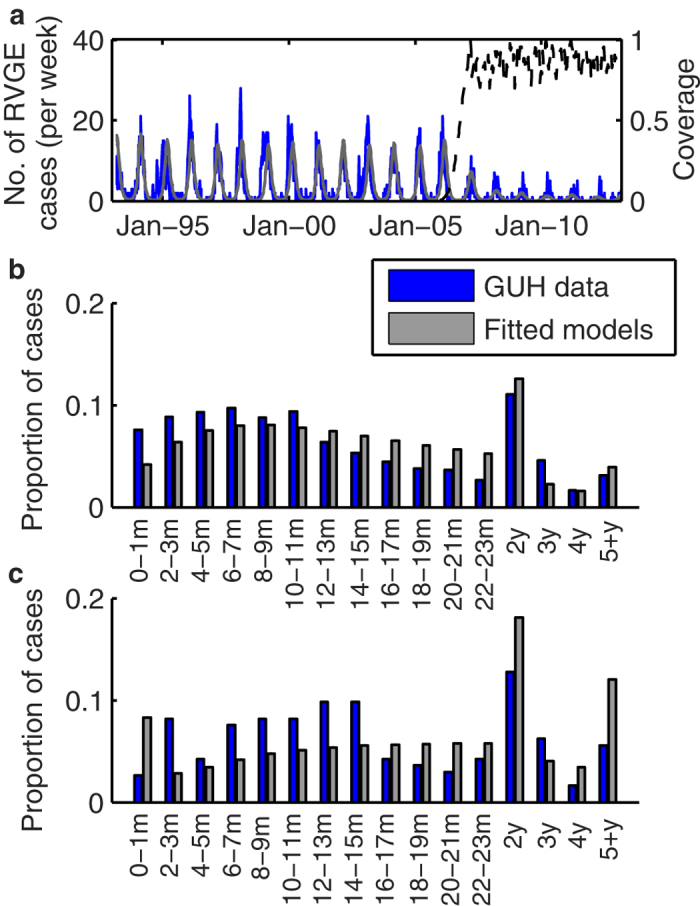
Validation of non-strain-specific model against hospitalization data from Gasthuisberg University Hospital (GUH) in Leuven, Belgium. (**a**) The number of weekly RVGE hospitalizations at GUH is plotted in blue, while the models fit to the Carenet-NCSF data then scaled by estimating the reporting fraction are plotted in grey. The black dashed line represents the vaccination coverage in Belgium. (**b**) The age distribution of RVGE patients at GUH (blue) and the mean age distribution predicted by the models (grey) prior to vaccine introduction (March 1999-June 2006). The black error bars represent the range of the fitted models. (**c**) The age distribution of RVGE patients at GUH (blue) and predicted by the fitted models (grey) following vaccine introduction (July 2006-June 2013).

We validated the non-strain-specific model by comparing to the hospitalization data from Gasthuisberg University Hospital (GUH) in Leuven, Belgium. The fitted models were able to capture both the size and timing of the rotavirus epidemic in most years, including the pre- and post-vaccination years not included in the Carenet-NCSF data (1993–2004, 2012–2013). However, we were not able to explain all of the year-to-year variability (e.g. the large peak in 1997/98 or the early activity in 1998/99) (Fig. 3a). Estimates of the reporting rate varied from 0.0155 to 0.0176 (Table 1). The models also provided a reasonably good fit to the age distribution of RVGE hospitalized cases (which included more detailed age classes than the data used for model fitting), although they tended to underestimate the post-vaccination number of hospitalizations in 2–15 month olds and overestimate the hospitalizations in the <2 month and ≥5 year age groups (Fig. 3b,c).

### Impact of vaccination on the distribution of rotavirus genotypes

#### Observed impact

**Figure 4:**
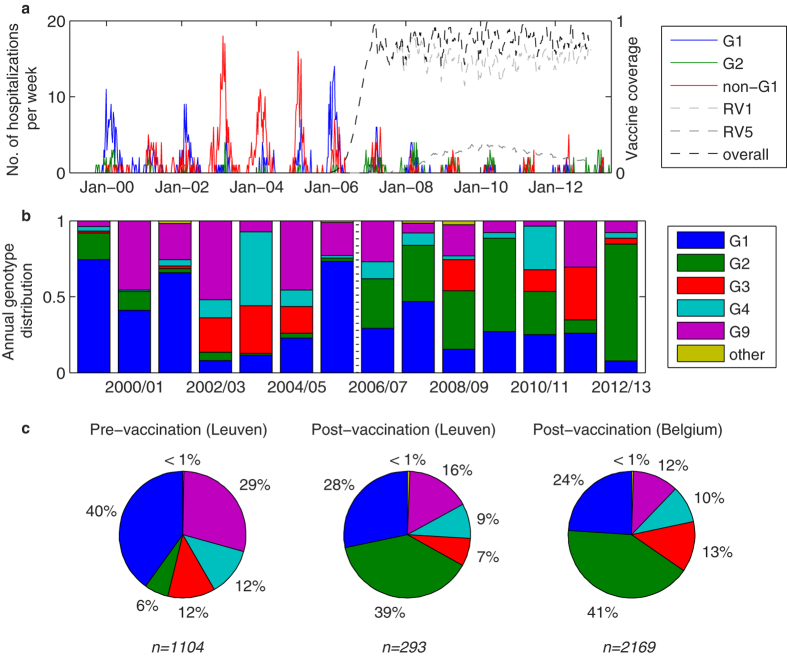
Distribution of rotavirus genotypes pre- and post-vaccination. (**a**) Number of rotavirus hospitalizations per week by genotype (G1—blue, G2—green, non-G1 P[8]—red) at GUH in Leuven, Belgium from September 1999 to June 2013. Overall vaccine coverage is indicated by the dashed black line, while the light grey line indicates coverage with the Rotarix vaccine (RV1) and the dark grey line represents coverage with RotaTeq (RV5). (**b**) Genotype distribution by year (July to June) from 1999/2000 to 2012/13. The dotted black line indicates the year of vaccine introduction. (**c**) Average genotype distribution over the seven seasons pre- (left) and post-vaccine introduction (middle) for RVGE hospitalizations at GUH in Leuven and for the Rotavirus Surveillance Network Belgium (right) from September 2007 to December 2012. The total number of typed samples (*n*) is listed under each pie.

There were a mean of 158 (range: 112–215) RVGE hospitalizations per year that were identified and typed at GUH in Leuven prior to vaccine introduction (Fig. 4a). On average 40% of these were G1, although this proportion varied from 8% (in 2002/03) to 74% (in 1999/00) (Fig. 4b). G9 was the second most prevalent G-type prior to vaccine introduction, responsible for 29% of hospitalizations overall (annual range: 4%–52%), while G2 was responsible for <18% of hospitalizations in any given year (6% overall) (Fig. 4b,c). Since January 2006, vaccination has been occurring primarily with the Rotarix (RV1) vaccine; coverage with RotaTeq (RV5) peaked at 19% in June 2010 (Fig. 4a). Following vaccine introduction, the proportion of hospitalizations due to G1 decreased to 28% overall in Leuven (range: 8–47%), while the proportion due to G2 jumped to 39% overall and 77% in 2012/13 (Fig. 4b,c). The post-vaccination genotype distribution for Belgium as a whole (based on data from the Rotavirus Surveillance Network Belgium (RSNB)) was similar (Fig. 4c). Other G-types (e.g. G12) accounted for <1% of all typed samples (Fig. 4c).

#### Parameter estimates

**Figure 5:**
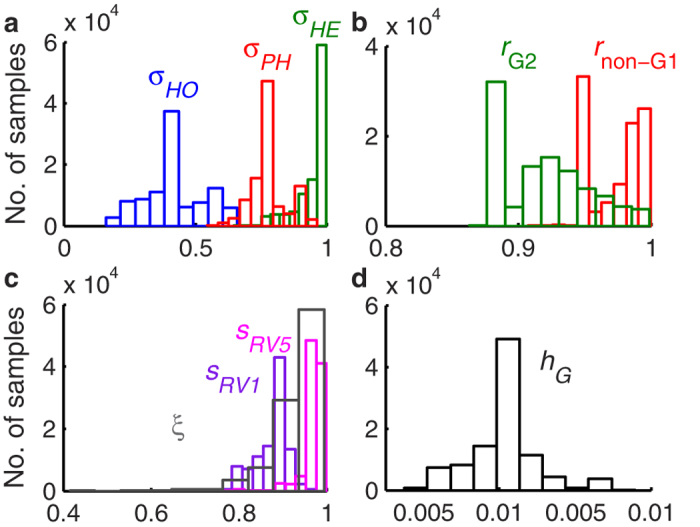
Posterior distribution of parameters estimated for the strain-specific model. Distribution of parameter estimates for (**a**) the relative risk of second infection with a homotypic strain (*σ*_*HO*,_ blue), partially heterotypic strain (*σ*_*PH*_, red), or fully heterotypic strain (*σ*_*HE*_, green) compared to the risk of first infection; (**b**) the relative infectiousness of non-G1 P[8] strains (*r*_1_, red) and G2 strains (*r*_*2*_, green) compared to G1 strains; (**c**) proportion of vaccinees who seroconvert and thus receive any benefit of vaccination with RV1 (*s*_*RV1*_, purple) or RV5 (*s*_*RV5*_, pink), and the proportion of vaccinees who receive broadly heterotypic protection equivalent to two natural infections (*ξ*, grey); and (**d**) the reporting fraction (*h*_*G*_, black) for moderate-to-severe RVGE cases in Belgium to be hospitalized and G-typed at GUH.

We estimated that heterotypic immunity (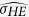 = 0.95, 95% credible interval (CI): 0.77–1.00) was considerably weaker than homotypic immunity (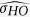 = 0.41, 95% CI: 0.20–0.62), while immunity against partially heterotypic strains was intermediate (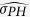 = 0.78, 95% CI: 0.65–0.91) (Fig. 5a). Both non-G1 P[8] strains (G3P[8], G4P[8] and G9P[8]) (*r*_1_ = 0.97, 95% CI: 0.95–1.00) and G2P[4] strains (*r*_2_ = 0.92, 95% CI: 0.88–0.99) were estimated to be slightly less transmissible than G1P[8] strains (Fig. 5b), which helps explain the predominance of G1P[8] prior to vaccine introduction^31^, although the 95% credible interval for the relative transmissibility of non-G1P[8] strains includes 1. Vaccination (with either vaccine) was estimated to generate a broadly heterotypic immune response in 94% (95% CI: 81–99%) of vaccinees (Fig. 5c). As such, the modelled vaccine effectiveness of RV1 against homotypic G1P[8] strains (*VE*_*RV1,HO*_ = 97.2%, 95% CI: 94.7–98.4%) versus partially heterotypic G3P[8], G4P[8], and G9P[8] strains (*VE*_*RV1,PH*_ = 95.1%, 95% CI: 90.9–97.1%) or fully heterotypic G2P[4] strains (*VE*_*RV1,HE*_ = 93.7%, 95% CI: 88.3–96.9%) did not vary substantially, and are similar to estimates of genotype-specific vaccine effectiveness from Belgium^29^,^30^. The modelled vaccine effectiveness for RV5 was similar albeit slightly higher (*VE*_*RV5*_ = 98.5%, 95%CI: 96.6–99.6%).

#### Predicted impact

**Figure 6:**
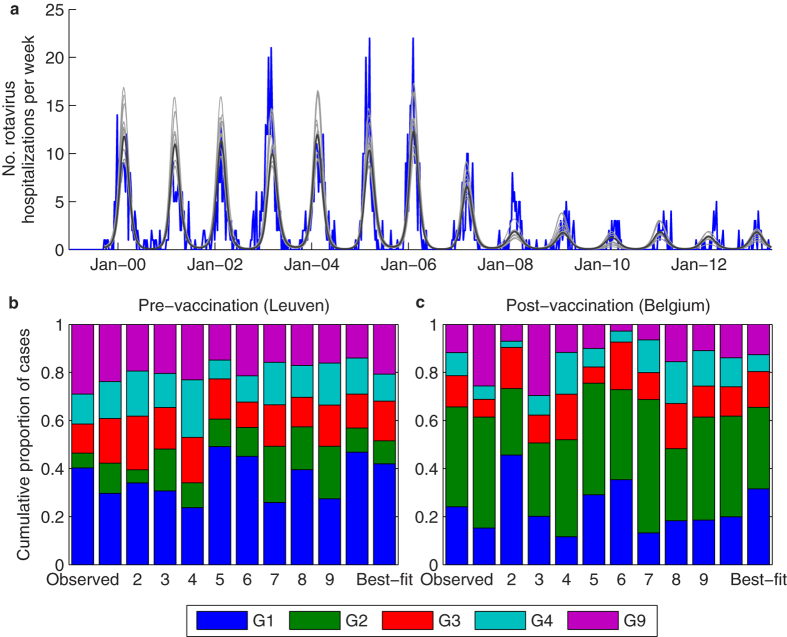
Model-predicted impact of vaccination on the incidence and genotype distribution of rotavirus in Belgium. (**a**) Number of typed rotavirus hospitalizations at GUH in Leuven (blue) and model-predicted number of rotavirus hospitalizations for 10 randomly sampled parameter sets from the posterior distribution (light grey) and the best-fit parameter set (dark grey). (**b**) Observed genotype distribution at GUH in Leuven from September 1999 to June 2006 and model-predicted pre-vaccination genotype distribution for 10 randomly sampled parameter sets and the best-fit model. (**c**) Observed genotype distribution from the Rotavirus Surveillance Network Belgium from September 2007 to December 2012 and model-predicted post-vaccination genotype distribution for 10 randomly selected parameter sets and the best-fit model.

These slight differences in immunity could explain the observed genotype patterns, including the increase in the proportion of RVGE hospitalizations caused by G2P[4] following vaccine introduction in Belgium (Fig. 6). The best-fit model was able to reproduce both the pre- and post-vaccination incidence of hospitalization for RVGE in Leuven, as well as the pre-vaccination distribution of genotypes in Leuven and the post-vaccination genotype distribution throughout Belgium (Fig. 6). When we sampled at random (without replacement) from the joint posterior distribution of the estimated parameters, all of the simulated models were able to capture the epidemic trajectories as well as differences between the pre- and post-vaccination genotype distributions (Fig. 6). However, the models had a tendency to slightly underestimate the size of epidemics in 2007/08, as well as some of the pre-vaccination seasons, while overestimating the size of other pre-vaccination epidemics (Fig. 6a). Furthermore, some models tended to slightly overestimate the proportion of cases due to G3P[8] and G4P[8] and slightly underestimate the proportion due to G9P[8] prior to vaccine introduction, whereas following vaccine introduction, the proportion of cases due to G1P[8] was slightly underestimated in some models and overestimated in others (Fig. 6b,c). It is likely that some of these discrepancies may be due to stochastic effects, which we do not account for in our model.

**Figure 7:**
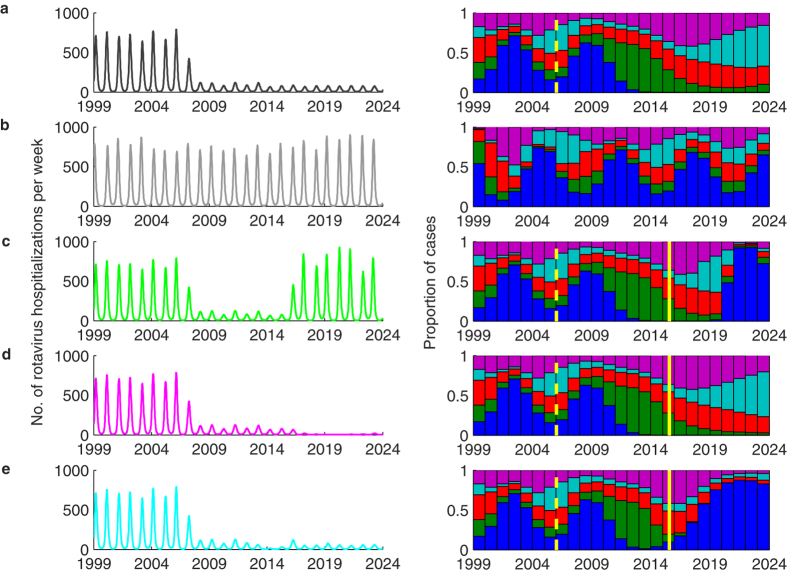
Model-predicted patterns of rotavirus incidence and annual genotype distributions. Predicted number of rotavirus hospitalizations per week in Belgium from January 1999 to December 2023 (left) and annual genotype distributions predicted by the model (right) are shown for the following scenarios: (**a**) best-fit model predictions given the current level of vaccine coverage; (**b**) the predicted incidence in the absence of vaccination; (**c**) vaccination at current levels, then no vaccination beginning in July 2015; (**d**) vaccination at current levels, then increasing to 100% coverage with two doses beginning in July 2015; (**e**) vaccination at current levels, but with 90% of vaccinated infants receiving RotaTeq and 10% receiving Rotarix beginning in July 2015. The dashed yellow lines in the plots of the annual genotype distribution represent the year of vaccine introduction, while the solid yellow lines represents July 2015. The colours correspond to the G-types as in previous figures (blue: G1, green: G2, red: G3; light blue: G4; purple: G9).

The best-fit model reproduces the observed cyclical pattern in the distribution of genotypes, although the timing of the cycles varies somewhat from the observed pattern (Fig. 7a). If vaccination coverage remains at the most recently observed levels, our model predicts that G2P[4] will remain the predominant genotype for another year or two at most, after which one or more of the non-G1 P[8] strains may predominate for a number of years (Fig. 7a). The prevalence of G1P[8] strains is predicted to decline to negligible levels in the near future, and the overall incidence of RVGE is expected to remain low (Fig. 7a). In the absence of vaccination, our model predicted that the incidence of RVGE would continue at the pre-vaccination levels, as would the cycling of genotypes, such that the average genotype distribution would remain similar to the pre-vaccination distribution over the 7-year timeframe between July 2006 and June 2013 (Fig. 7b). If vaccination were to stop in July 2015, the incidence of RVGE is expected to rebound to pre-vaccination levels within two years, and G1P[8] is expected to re-emerge as the predominant strain within five years (Fig. 7c). If coverage were to increase to 100% with at least two doses of the vaccines, the incidence of RVGE is expected to decline to very low levels, with <500 hospitalizations per year in Belgium (Fig. 7d). Finally, if vaccination were to continue at current levels overall, but market shares of Rotarix and RotaTeq were to switch such that 90% of all vaccines administered were RotaTeq, the incidence of RVGE would remain similar to the current post-vaccination levels, but G1P[8] would be expected to become the predominant strain within a few years (Fig. 7e).

## Discussion

This is the first study to use a mechanistic model of vaccine impact to show that observed changes in the rotavirus genotype distribution in Belgium, in particular the prolonged increased prevalence of G2P[4] strains, can be attributed at least in part to the pressures imposed by large-scale vaccination. While vaccine effectiveness has been shown to be similarly high against all the circulating rotavirus genotypes, consistent with our model estimates, slight differences in homotypic versus heterotypic immunity against second infection can explain both the observed cycling of genotypes as well as the post-vaccination changes in the genotype distribution. Overall, however, the incidence of hospitalization due to RVGE is expected to remain low with continued vaccination.

We used a hierarchy of models, beginning with a simplified model that does not distinguish between the different genotypes of rotavirus (non-strain-specific model), which we showed could explain the observed impact of vaccination on the size and timing of peaks of rotavirus hospitalizations in Belgium, as well as the age distribution of hospitalized cases. We validated this model by showing that it could also explain the observed number and age distribution of RVGE hospitalizations at GUH in Leuven from March 1999 to June 2013. However, the models had a tendency to slightly underestimate the proportion of 6–15 month old patients and slightly overestimate the proportion of patients <2 months of age and older children and adults post-vaccination. This may be due to our assumptions of homogeneous mixing and/or exponential waning of maternal immunity, while the discrepancy between the observed and model-predicted hospitalizations among ≥5 year olds could be due to reporting biases or model misspecification.

We found that the models had a tendency to predict multi-annual and chaotic dynamics, particularly following vaccine introduction. This has also been exhibited by other rotavirus models^34^. Under certain parameter combinations, the reduction in transmission associated with large-scale vaccination could lead to a period of years in which very few infections occurred, followed by larger outbreaks. While small annual outbreaks of rotavirus have been occurring in Belgium since vaccine introduction in 2006, the United States has been exhibiting biennial post-vaccination epidemics^5^. Belgium is a small country surrounded by countries that have yet to introduce rotavirus vaccination into their routine immunization schedules (at least until recently). It is possible that the transmission pressure exerted by the surrounding countries has had a stabilizing influence on the post-vaccination dynamics of rotavirus in Belgium, leading to annual epidemics and the continued circulation of G1P[8] strains. Likewise, it has been hypothesized that rotavirus vaccination in Belgium and other neighbouring countries may have contributed to the exceptionally low rotavirus incidence in the Netherlands (which has yet to introduce vaccination) in 2013/14^35^. Our model predicts that G1P[8] strains could be essentially eliminated from Belgium in the near future if vaccination were to continue at current levels. However, we did not account for possible re-introduction of G1P[8] strains from surrounding countries, nor did we account for within-genotype evolution, which may lead to selection for G1P[8] strains that escape homotypic immunity from the vaccine strain^36^. G1P[8] continued to be the predominant genotype observed in France, the United Kingdom, and other western European countries between 2007–2013^37^.

The predominance of G2P[4] and other fully heterotypic genotypes (e.g. G8P[4]) has also been observed in other countries that have introduced the Rotarix vaccine, including Austria, Brazil, and some Australian states^20^,^25^,^26^,^27^,^37^,^38^,^39^. In Brazil, G2P[4] was the predominant genotype from 2006–2011, with prevalence ≥50% in all years except 2009, while G1P[8] has been relatively rare^18^,^27^. Furthermore, another fully heterotypic genotype (G8P[4]) emerged in Northeast Brazil in 2012^39^. However, a high prevalence of G2P[4] strains was also observed in other South and Central American countries without an established rotavirus vaccination program around the same time^38^,^40^. While it is not unusual for G2P[4] to be the predominant genotype for a year or two in the absence of vaccination, our analysis suggests that vaccination may have contributed to its continued high relative prevalence in Belgium over the seven years since vaccine introduction.

Our use of semi-nested models, beginning with the non-strain-specific model, then extending it to differentiate among the five major rotavirus genotypes, was necessary because having information on the age distribution of cases is essential for informing estimates of the basic reproductive number, *R*_0_, but including age structure in the strain-specific model considerably increases the computational burden, making the model fitting process too time-consuming. The range of *R*_0_ estimates from the fitted models were similar to those generated by different models fit to pre-vaccination data from England and Wales^41^. Various models have assumed either no complete immunity following infection or temporary immunity lasting ≤1 year. However, in preliminary analyses, we noted that a longer duration of immunity (in the range of 6–12 months) was necessary to explain the observed cycling of genotypes in the strain-specific model.

We had to make a number of simplifying assumptions to limit the number of estimated parameters to an identifiable subset. In particular, we assumed that the infectiousness and severity of second infections caused by homotypic versus heterotypic strains was proportional to the relative risk of infection, and that the non-G1 P[8] strains were equally transmissible. Furthermore, we assumed that the duration of complete immunity following vaccination was the same as following natural infection; thus, we assumed some partial waning of vaccine-induced immunity, but did not attempt to estimate its duration, as others have done^32^,^34^.

It is difficult for the model to capture the exact genotype distributions through time because they are subject to stochastic variation as well as cyclical fluctuations. Therefore, we focused our efforts on being able to capture the average genotype pattern over the seven years before and six years after vaccine introduction (which is approximately equal to the multi-annual period of oscillations^31^), as well as the overall number of RVGE hospitalizations by week. We have shown previously that small amounts of stochastic variation (by allowing for fadeout and reintroduction of genotype-specific infections) helped to reproduce some of the observed short-term patterns, but the average long-term patterns remained qualitatively the same. These stochastic effects could help to explain why G9 strains were more prevalent than G3 and G4 strains in our data from Leuven. Furthermore, evidence suggests that G9P[8] strains have emerged more recently than the other common rotavirus genotypes^42^. While we attempted to account for this by introducing G9 infections part way through the burn-in period in our model (based on estimates of the time to most recent common ancestor), the precise timing of introduction into the local population is difficult to determine.

Nevertheless, we believe our analysis is robust. The estimated variation in homotypic versus heterotypic immunity can help to explain the observed changes in the distribution of rotavirus genotypes in Belgium, where vaccination has been occurring primarily with the monovalent Rotarix vaccine since 2006. Furthermore, our fitted model can be used to predict long-term trends in the genotype distribution and explore potential scenarios related to vaccination policy. We believe our models are generalizable to other high-income countries as well, although we expect there will be slight differences in the transmission rate of rotavirus from country to country^41^,^43^,^44^. Our finding that vaccination is estimated to provide broadly heterotypic immunity suggests that the greater diversity of rotavirus genotypes typical of developing countries is likely not the primary reason for the reduce vaccine efficacy observed in low-income settings, consistent with observations from clinical trials and vaccine effectiveness studies^45^,^46^,^47^,^48^. Models that take into account the apparent differences in natural and vaccine-induced immunity between developed and developing countries are needed to better understand rotavirus dynamics and the impact of vaccination in low-income settings.

## Materials and Methods

### Data sources

We utilized national and local-level data to assess the epidemiological impact of rotavirus vaccination in Belgium. At the national level, data were obtained on the weekly number of hospitalized patients with a rotavirus diagnosis from July 2004 to June 2012 from members of the National Alliance of Christian Sickness Funds (NCSF) through the Carenet database. Carenet is designed for the exchange of information about hospitalizations between hospitals and all health insurance companies in Belgium through an electronic system. From Carenet, information was obtained from the members of only one health insurance company (NCSF), which is however the largest—it provides health insurance for approximately 42% of the Belgian population. Data on any patient for which the diagnosis contained one of the following search strings were included: ‘rota’ or ICD-9-CM code ‘008.61’ or ICD-10 code ‘A08.0’. A medical clinician searched the diagnostic fields of the retrieved records manually and selected the ones for which rotavirus was likely the main reason for hospitalization. The age of the patients was available for 6-month age groups from 0 to 2 years of age, 1-year age groups from 2 to 5 years of age, a 5–10 year old age group, and ≥10 years of age. All data extractions and associated analyses related to NCSF were performed at the Medical Management Department of the NCSF under the supervision of the Chief Medical Officer. The other research partners received no personally identifiable information (including small cells) from NCSF. We also corrected for the changing coverage of NCSF and the Carenet database over time: the proportion of the Belgian population belonging to NCSF decreased slightly between 2004 and 2012 (Fig. 1a), whereas during this time, coverage of the Carenet database increased from only 7% of hospital beds in Belgium in January 2004 to 22% by July 2004 to nearly 100% of hospital beds by 2009 (Fig. 1a). Model output was multiplied by the proportion of the population belonging to NCSF and the proportion of hospital beds covered by the Carenet database when fitting the model to the observed data.

At the local level, we had data on genotype-specific hospitalizations for RVGE among patients presenting to the Gasthuisberg University Hospital (GUH) in Leuven, Belgium. The incidence of hospitalized RVGE cases at GUH has been recorded since January 1981, and rotavirus positive samples have been G-typed since September 1999 and G/P-typed since 2003^28^,^49^,^50^. Prior to 1993, only data on the total monthly number of hospitalization was available. We extracted the data available from January 1993 through June 2013 and aggregated the number of hospitalized RVGE cases according to the week of admission, age of patient (in 2-month age groups from 0–24 months, 1-year age groups from 2 to 4 years, and ≥5 years of age), and genotype (G1P[8], G2P[4], G3P[8], G4P[8], G9P[8], other).

We also had post-vaccination data from the Rotavirus Surveillance Network Belgium (RSNB) from September 2007 to June 2012. This network receives rotavirus positive stool samples (sent on a voluntary basis) from hospitals, diagnostic centres, and individual paediatricians throughout Belgium. Under the framework of this network, a total of 2,169 samples were genotyped between September 2007 and June 2012 (≈400–600 samples per rotavirus season).

To assess changes in vaccine coverage, we used data on (1) sales of the Rotarix and RotaTeq vaccines in Belgium from GSK and Sanofi Pasteur MSD, and (2) reimbursements for rotavirus vaccines from the Inter Mutualistic Agency (IMA-AIM) (for details, see^33^). Since Belgium did not begin reimbursing individuals for rotavirus vaccines until October 2006, we used the sales data to inform the vaccination coverage rate up until February 2007 (Fig. S1). Sales of RotaTeq in Belgium did not begin until after February 2007. We used the IMA-AIM data to inform the coverage estimates from February 2007 to December 2012, although the two data sources were largely in agreement for 2007–2009 (Fig. S1). We calculated the vaccination coverage by first smoothing the data on the number of infants vaccinated per week by calculating a 5-week moving average. We then divided the number of children vaccinated with at least one dose in week *w* by the number of births in week *w*-8, which was interpolated from data on monthly births in Belgium (http://ec.europa.eu/eurostat/data/database). To calculate coverage with two/three doses (Rotarix/RotaTeq), we likewise divided the number of fully vaccinated children by the number of births in week *w*-12. We assumed coverage in January-June 2013 remained at its most recently observed level (from December 2012).

### Description of the models

We used two *SIR*-like compartmental models for the transmission dynamics of rotavirus to describe the incidence of RVGE—a simplified model in which we do not distinguish among the possible genotypes causing infection (non-strain-specific model) and a more complex model in which we differentiate between infection (and previous infection) with five different rotavirus strains, representing the five major rotavirus genotypes (strain-specific model) (Fig. 2). Both models have been described previously^31^,^41^,^44^, although we made some minor modifications as described below. More details can be found in the Supplementary Methods.

Briefly, both models assume that individuals are born with maternal immunity (*M*) (which provides equal protection against all rotavirus strains) that wanes after a mean duration of 1/*ω*_*M*_ = 13 weeks^51^, leaving the infant susceptible to a first rotavirus infection (*S*_0_). For the non-strain-specific model (Fig. 2a), we assume first infection (with any rotavirus genotype) occurs at a rate *λ*(*t*). Infected individuals (*I*_1_) are infectious for an average of 1/*γ*_1_ = 1 week^52^,^53^, after which we assume they are temporarily immune to reinfection (*R*_1_) for a period of 1/*ω* weeks (estimated). Following this period of temporary immunity, the individual becomes susceptible to a second infection (*S*_1_), which occurs at a reduced rate *σ*_1_*λ*(*t*)^54^. Second infections (*I*_2_) are assumed to be less infectious (*ρ*_2_ = 0.5)^55^,^56^, of shorter duration (1/*γ*_2_ = 0.5 week)^57^,^58^,^59^, and less likely to result in severe RVGE compared to first infections^54^. Again, infectious individuals recover and are temporarily immune to reinfection (*R*_2_). Once this immunity wanes, individuals become susceptible to third (and subsequent) infection (*S*_*A*_), which occur at a further reduced rate *σ*_2_*λ*(*t*)^54^. We assume that these subsequent infections (*I*_*A*_) are only mildly symptomatic (i.e. are not associated with severe RVGE (*D*) and hence are not reported in our datasets)^54^ and are less infectious (*ρ*_A_, estimated) and of shorter duration (1/*γ*_2_ = 0.5 week) compared to first infections. They are again followed by a period of temporary immunity (*R*_*A*_); here, we make the simplifying assumption that this immunity wanes at the same rate *ω* as estimated previously, and individuals return to the *S*_*A*_ state.

For the strain-specific model (Fig. 2b), we differentiate between first infection with genotype *g* = 1 to 5 (where *g* = 1 corresponds to G1P[8], *g* = 2 corresponds to G2P[4], *g* = 3 corresponds to G3P[8], *g* = 4 corresponds to G4P[8], and *g* = 5 corresponds to G9P[8]). Fully susceptible individuals (*S*_0_) are infected with genotype *g* at a rate *λ*_*g*_(*t*) and enter infected state *I*_*P,g*_ and remain infectious for an average of 1/*γ*_1_ = 1 week. Again, we assume infection is followed by a short period of temporary immunity against all strains (*R*_*g*_), which wanes at a rate *ω*, leaving the individual susceptible to second infection (*S*_*g*_). We keep track of the genotype *g* causing first infection because we need to differentiate between second infection occurring with the same genotype (at a rate *σ*_*HO*_*λ*_*g*_(*t*)), versus a partially heterotypic (at a rate *σ*_*PH*_*λ*_*j≠g*_(*t*)) or fully heterotypic genotype (at a rate *σ*_*PH*_*λ*_*j≠g*_(*t*)). We assume that both the relative infectiousness and severity of second infections with a homotypic (*I*_*HO,g*_) versus partially or fully heterotypic genotype (*I*_*HE,g*_) are proportional to *σ*_*HO*,_ *σ*_*PH*_, and *σ*_*HE*_, respectively, but that all second infections have the same duration of infectiousness, 1/*γ*_2_ = 0.5 week. This reduces the number of free parameters to be estimated to only those in which we are most interested and have limited data to inform. Following infection with any two genotypes, we assume that individuals develop a broad cross-protective immunity to all genotypes^31^,^60^. As in the non-strain-specific model, individuals are temporarily immune to reinfection (*R*_*A*_), then become susceptible to subsequent infections at a reduced rate *σ*_2_*λ*_*g*_(*t*). Subsequent infections with genotype *g* are again only mildly symptomatic and shorter and less infectious compared to first infections. Thus, again we assume that only first and second infections can result in moderate-to-severe RVGE with genotype *g* (*D*_*g*_). The proportion of first infections that result in severe RVGE was again assumed to be *d*_1_ = 0.13 for all genotypes^54^, while the proportion of second infections resulting in moderate-to-severe RVGE was assumed to vary for homotypic versus heterotypic strains with a (weighted) mean equal to *d*_2_, such that *d*_2,*HO*_ = *d*_2_*σ*_*HO*_/*σ*_2_, *d*_2,*HE*_ = *d*_2_*σ*_*HE*_/*σ*_2_, and *d*_2,*PH*_ = *d*_2_*σ*_*PH*_/*σ*_2_.

The force of infection with genotype *g* at time *t* in weeks (*λ*_*g*_(*t*)) is given by:


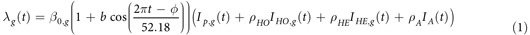


where *β*_0,*g*_ is the baseline transmission parameter for genotype *g*, *b* is the amplitude of seasonality in the transmission rate, and *ϕ* is the seasonal offset parameter (i.e. timing of peak transmission, in weeks). We assume that the transmissibility of the non-G1 P[8] strains is reduced by a factor *r*_1_ and the transmissibility of G2P[4] strains is reduced by *r*_2_ relative to G1P[8], such that *β*_0,3_ = *β*_0,4_ = *β*_0,5_ = *r*_1_*β*_0,1_ and *β*_0,2_ = *r*_2_*β*_0,1_. This was necessary to explain why G1P[8] was the predominant genotype prior to vaccine introduction, as described previously^31^.

Note that the non-strain-specific model is nested within the strain-specific model; however, we incorporate age structure in the non-strain-specific model but not the strain-specific model. Given the same age structure, both models should yield the same overall dynamics when *r*_1_ = *r*_2_ = 1 and *σ*_*HO*_ = *σ*_*PH*_ = *σ*_*HE*_ = 0.62. Thus, the weighted average of *σ*_*HO*_, *σ*_*PH*_, and *σ*_*HE*_ should be approximately equal to *σ*_1_ = 0.62; however, we do not know the weights, which vary with time and correspond to the likelihood of being exposed to someone who is infectious with a homotypic versus partially or fully heterotypic genotype.

### Modeling vaccination

We assume that a fraction *s*_*RV1*_ and *s*_*RV5*_ seroconvert following vaccination with Rotarix and RotaTeq, respectively. In the non-strain-specific model, we assume that one dose of vaccine confers immunity comparable to one natural infection, thereby moving individuals from the *M* and *S*_0_ compartments into the *R*_1_ compartment (Fig. 2a). Vaccination occurs upon aging into the 2-month age class (first dose) and 4-month age class (second dose). A second (and third) dose of vaccine confers additional immunity comparable to a second natural infection in a fraction *ξ* of fully vaccinated individuals, thereby moving individuals from the *R*_1_ or *S*_1_ state into the *R*_2_ state (Fig. 2a).

In the strain-specific model, we model vaccination with Rotarix and RotaTeq separately. Upon birth (since we do not include age structure), vaccinated individuals enter either the *V*_*R*,*RV1*_ or *V*_*R*,*RV5*_ compartment, depending on the vaccine coverage (Fig. 2b). A fraction *ξ* who is fully vaccinated with either vaccine can develop a broadly heterotypic immunity (comparable to two natural infections) and enter the *R*_*A*_ state. We assume a fraction *s*_*RV1*_ and *s*_*RV5*_ who seroconverted following vaccination (estimated, see Table 2) are temporarily immune, with the duration of immunity comparable to that following natural infection (1/*ω*). Once this immunity wanes, vaccinated individuals are susceptible to infection (*V*_*S*,*RV1*_ or *V*_*S,RV5*_), but at a reduced rate depending on the type of immunity conferred by the vaccine. We assume that the RV1 vaccine confers homotypic immunity against G1P[8], heterotypic immunity against G2P[4], and partially heterotypic immunity against the non-G1 P[8] strains, whereas for simplicity we assume that RV5 confers homotypic immunity against all five strains (Fig. 2b).

We can calculate the modelled vaccine effectiveness as:


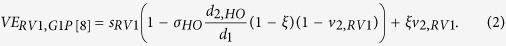



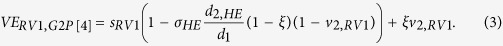



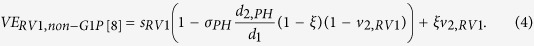



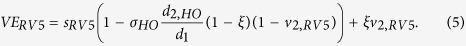


where *v*_2,*RV1*_ and *v*_2,*RV5*_ are the mean proportion of fully vaccinated individuals with Rotarix and RotaTeq vaccines, respectively.

*Modelling the impact of vaccination on rotavirus hospitalizations in Belgium.* 

We began by fitting the non-strain-specific age-structured model (Fig. 2a) to data on hospitalizations due to rotavirus from the Carenet-NCSF database (Fig. 1a). We generated 10,000 parameter sets for the eight parameters to be estimated, sampling from reasonable ranges for each parameter using Latin Hypercube Sampling (LHS) (Table 1). We simulated the incidence of severe RVGE in Belgium and calculated the log-likelihood of the data given the model, assuming that the number of hospitalizations reported in the dataset were Poisson distributed with a mean equal to the model-predicted incidence of severe RVGE (*D*_*a,w*_) times a hospitalization/reporting factor (*h*_*a*_, estimated) and a correction factor that accounts for changes in the coverage of the Carenet database over time and the proportion of the Belgian population covered by NCSF (*c*):


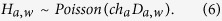


(see Supplementary Methods). We allowed the reporting factor *h*_*a*_ to differ between children <2 years of age and older children and adults, with the reporting factor in 2-year olds equal to the mean of these two reporting factors, in order to account for differences in testing and diagnosis rates, since the cost of the rotavirus test is only reimbursed for children <2 years of age in Belgium (since 2004). We assumed homogeneous mixing for simplicity and because we did not include age structure in the strain-specific model, since doing so caused the model to run prohibitively slowly. We assumed uniform prior distributions for all estimated model parameters over ranges that were either uninformative or consistent with previous model estimates^41^, with the exception of the duration of temporary immunity (Table 1). We constrained 1/*ω* to be between 26 and 52 weeks, since we noted that a longer duration of temporary immunity was necessary to reproduce the observed cycling of genotypes in strain-specific model.

We then used the 10 parameter sets that yielded the highest posterior probability as initial conditions for a simplex search algorithm to identify local maxima in the posterior probability surface. We used “fminsearch” in MATLAB v7.14 (MathWorks, Natick, MA) to minimize the negative log posterior probability. Multiple different parameter sets provided an approximately equally good fit to the data (Table 1). Since our priority here lies in understanding the impact of vaccination on the distribution of rotavirus genotypes, we proceeded using the parameter set yielding the highest posterior probability for the strain-specific model, then examined the sensitivity of our conclusions to this choice (see Supplementary Methods).

We validated the non-strain-specific model by comparing the fitted models to the hospitalization data from GUH in Leuven, Belgium. The only parameter we allowed to vary was the reporting rate for <2 year olds (since the population who seek care at GUH is some unknown fraction of the total Belgian population), which we estimated by fitting the model output to the GUH data from March 1993 to December 2013.

### Modelling the impact of vaccination on the distribution of rotavirus genotypes

We identified nine key parameters to be estimated for the strain-specific model (Fig. 2b): the relative infectiousness of the non-G1 P[8] strains compared to G1P[8] (*r*_1_); the relative infectiousness of G2P[4] compared to G1P[8] (*r*_2_); the relative risk of second infection with a homotypic strain (same G- and P-type as the strain causing first infection) (*σ*_*HO*_); the relative risk of second infection with a partially heterotypic strain (different G-type, same P-type) (*σ*_*PH*_); the relative risk of second infection with a fully heterotypic strain (different G- and P-type) (*σ*_*HE*_); the proportion of fully vaccinated individuals who develop a broadly heterotypic immune response (*ξ*); the proportion of those vaccinated with at least one dose of RV1 who seroconvert and therefore receive any protection (*s*_*RV1*_); the proportion of those vaccinated with at least one dose of RV5 who seroconvert (*s*_*RV5*_); and the reporting fraction for severe RVGE cases presenting to GUH (*h*_*G*_). We assumed uniform prior distributions for all parameters with the exception of *s*_*RV1*_ and *s*_*RV5*_, for which we fitted beta prior distributions to the mean and variance of the rate of seroconversion to each vaccine in low child mortality countries reported in a recent meta-analysis (Supplementary Table S1)^61^.

We started by generating 100,000 parameter sets by sampling from reasonable parameter ranges for the nine parameters to be estimated using LHS (Supplementary Table S1; see Supplementary Methods). We then simulated the strain-specific model under each of the sampled set of parameters and evaluated the ability of the model to reproduce three key features of the data: (1) the observed weekly time series of RVGE hospitalizations at GUH from September 2007-June 2012; (2) the pre-vaccination distribution of the five common genotypes among GUH patients; and (3) the post-vaccination genotype distribution for the RSNB. We again assumed that the weekly number of RVGE cases as GUH was Poisson-distributed with a mean equal to the model-predicted incidence of severe infection times the reporting fraction *h*_*G*_. To calculate the likelihood contribution of the pre- and post-vaccination genotype distributions, we assumed the observed total number of infections with each genotype followed a multinomial distribution with probabilities given by the model-predicted genotype distribution. We scaled each of these components such that they contributed approximately equally to the overall likelihood in order to ensure that the model was able to replicate all three features of the data (see Supplementary Methods).

We used the 10 parameter sets with the highest posterior probability to initialize a simplex search for the best-fit parameters for the strain-specific model, but again identified multiple parameter sets with approximately equal support (see Supplementary Methods; Supplementary Table S2). We therefore adopted an approach in which we used importance sampling to approximate the posterior distribution of the parameters of interest for the strain-specific model, *f*(***θ***_***S***_). For our sampling distribution, *g*(***θ***_***S***_), we used a multivariate normal distribution on the logit scale, with a mean equal to logit 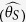, where 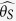 corresponds to the parameter set with the highest posterior probability identified from the simplex search, and variance equal to two times the variance-covariance matrix of the logit-transformed top 100 parameter sets. We generated *N* = 100,000 samples from *g*(***θ***_***S***_) and calculated the log-likelihood of the model under each of the parameter sets. We then weighted each of parameter set by:


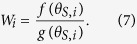


We then resampled (with replacement) from *g*(***θ***_***S***_) according to these weights to generate the posterior distribution and calculated the mean of ***θ***_***S***_ as follows:


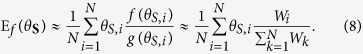


The 95% credible interval (CI) for ***θ***_***S***_ was estimated from the 2.5^th^ and 97.5^th^ percentiles of the posterior distribution.

## Additional Information

**How to cite this article**: Pitzer, V. *et al.* Did Large-Scale Vaccination Drive Changes in the Circulating Rotavirus Population in Belgium? *Sci. Rep.* **5**, 18585; doi: 10.1038/srep18585 (2015).

## Supplementary Material

Supplementary Information

## Figures and Tables

**Table 1:** Estimated parameters for the non-strain-specific model fit to the Carenet-NCSF data.

Parameter	Symbol	Initial range	Prior dist-ribution	Model 1	Model 2	Model 3	Model 4	Model 5	Model 6	Model 7	Model 8	Model 9	Model 10
Basic reproductive number	*R*_0_ = *β*_0_/*γ*	15 to 30	U (5,30)	20.0	17.3	15.5	19.5	15.1	20.9	13.6	21.6	17.3	15.8
Relative infectiousness of mild/asymptomatic infections	*ρ*_*A*_	0.1 to 0.3	U (0,0.5)	0.16	0.19	0.23	0.16	0.24	0.11	0.29	0.12	0.19	0.23
Amplitude of seasonality in transmission	*b*	0.05 to 0.1	U (0,0.3)	0.095	0.086	0.084	0.094	0.085	0.089	0.081	0.095	0.086	0.084
Seasonal offset	*ϕ*	−0.03 to 0.07 years	U (−0.5,0.5)	−0.022	0.020	0.011	0.008	−0.028	0.015	0.008	−0.004	0.009	0.005
Duration of temporary immunity	1/*ω*	26 to 39 weeks	U (26,52)	28.4	32.4	31.3	31.4	26.5	30.5	30.9	31.9	35.0	35.9
Proportion of vaccinees receiving protection equal to two natural infections	*ξ*	0.2 to 0.8	U (0,1)	0.48	0.59	0.37	0.90	0.71	0.29	0.85	0.43	0.89	0.98
Hospitalization/reporting rate among < 2 year olds	*h*_0_	0.48 to 0.52	U (0,1)	0.52	0.51	0.43	0.50	0.48	0.50	0.44	0.52	0.55	0.53
Relative rate of hospitalization among ≥ 3 year olds	*η*	0.2 to 0.6	U (0,1)	0.28	0.20	0.21	0.23	0.23	0.20	0.25	0.31	0.15	0.15
Log posterior				−5,191	−5,290	−5,218	−5,112	−5,243	−5,366	−5,236	−5,191	−5,137	−5,098
*Model validation*													
Hospitalization/reporting rate among < 2 year olds at GUH	*h*_*G*_	NA	U (0,1)	0.0155	0.0168	0.0164	0.0163	0.0164	0.0171	0.0163	0.0155	0.0176	0.0175
Log posterior				−7,288	−7,267	−7,271	−7,273	−7,271	−7,265	−7,273	−7,287	−7,264	−7,264
