# Supplementary material for: Did Large-Scale Vaccination Drive Changes in the Circulating Rotavirus Population in Belgium?
Source: Sci Rep. 2015 Dec 21;5:18585. doi: 10.1038/srep18585 (PMC4685644; doi:10.1038/srep18585)
Supplement: Supplementary Information [file srep18585-s1.pdf]

Did Large-Scale Vaccination Drive Changes in the Circulating Rotavirus Population in Belgium?

Virginia Pitzer*[1,2], Joke Bilcke[3], Elisabeth Heylen[4], Forrest W. Crawford[5], Michael Callens[6], Frank De Smet[6,7], Marc Van Ranst[4], Mark Zeller[4], and Jelle Matthijnssens*[4]

[1] Department of Epidemiology of Microbial Diseases, Yale School of Public Health, New Haven, Connecticut, United States of America;

[2] Fogarty International Center, National Institutes of Health, Bethesda, Maryland, United States of America;

[3] Centre for Health Economics Research & Modeling of Infectious Diseases (CHERMID), Vaccine and Infectious Disease Institute (VAXINFECTIO), University of Antwerp, Wilrijk, Antwerp, Belgium;

[4] KU Leuven - University of Leuven, Department of Microbiology and Immunology, Laboratory for Clinical and Epidemiological virology, Rega Institute for Medical Research, Leuven, Belgium;

[5] Department of Biostatistics, Yale School of Public Health, and Department of Ecology and Evolutionary Biology, Yale University, New Haven, Connecticut, United States of America;

[6] National Alliance of Christian Sickness Funds, Brussels, Belgium;

[7] KU Leuven – University of Leuven, Department of Public Health and Primary Care, Environment and Health, Leuven, Belgium

* Corresponding authors:
Jelle Matthijnssens, Rega Institute for Medical Research, Minderbroedersstraat 10, B-3000 Leuven, Belgium. Tel: +32 16 332166, Fax: +32 16 33 21 31, E-mail: jelle.matthijnssens@uz.kuleuven.ac.be
Virginia Pitzer, Department of Epidemiology of Microbial Diseases, Yale School of Public Health, New Haven, CT 06520-8034 USA. Tel: +1-203-785-5053, E-mail: virginia.pitzer@yale.edu

Supplementary Methods

Initial conditions

We initialized the models assuming there was one primary infection in each age class (except for the <2 month old age group), or 20 primary infections with each of the G1-G3 strains for the strain-specific model, beginning in the first week of January 1970. All other individuals in the population were assumed to be fully susceptible, with the exception of the <2 month olds, who we assumed began in the *M* state. In the strain-specific model, we introduced a single primary infection with G4 in January 1971 and a single primary infection with G9 in January 1989, to allow for some differentiation among the non-G1 P[8] strains and in line with evidence of the more recent emergence of G9P[8]. We then ran the model until quasi-equilibrium, which appeared to have been reached by the start of our datasets (which varied from January 1993 for the GUH data to July 2004 for the Carenet-NCSF data). Hence, the model "burn-in period" was 23 years when comparing model output to the GUH non-strain-specific data, 29.7 years for the GUH strain-specific data, and 34.5 years for the Carenet-NCSF data.

Model fitting procedure

We calculated the model-predicted incidence of severe RVGE ($D_{a,w}$) for the non-strain-specific model as:

$$D_{a,w} = \int_{t=w-1}^{w} \lambda(t)\left(d_1 S_{0,a}(t) + d_2 \sigma_1 S_{1,a}(t)\right) dt \tag{1}$$

where

$$\lambda(t) = \beta_0 \left(1 + b\cos\left(\frac{2\pi t - \phi}{52.18}\right)\right)\left(I_1(t) + \rho_S I_2(t) + \rho_A I_A(t)\right) \tag{2}$$

The log-likelihood of the model was calculated by assuming that the number of reported hospitalizations was Poisson distributed with a mean equal to the model-predicted incidence of severe RVGE ($D_{a,w}$) times a hospitalization/reporting factor (h_a, estimated) and a correction factor that accounts for changes in the coverage of the Carenet database over time and the proportion of the Belgian population covered by NCSF (c_w):

$$LL = \sum_w \sum_a \left(H_{a,w} \log(c_w h_a D_{a,w}) - c_w h_a D_{a,w} - \sum_{i=1}^{H_{a,w}} \log(i) \right) \tag{3}$$

We allowed the reporting factor h_a to differ between children <2 years of age and older children and adults, with the reporting factor in 2-year olds equal to the mean of these two reporting factors, in order to account for differences in testing and diagnosis rates.

For the strain-specific model, we evaluated the model fit based on the ability of the model to reproduce three components of the data: (1) the observed weekly time series of RVGE hospitalizations at GUH from September 2007-June 2012; the pre-vaccination distribution of the five common genotypes among GUH patients; and the post-vaccination genotype distribution for the RSNB. We calculated the log-likelihood of the time series (LL_{ts}) assuming that the total number of RVGE cases each week at GUH ($G_w = \sum_g G_{w,g}$) was Poisson-distributed with a mean equal to the model-predicted number of severe RVGE infections with all genotypes g ($D_w = \sum_g D_{w,g}$) times the reporting fraction h_G:

$$LL_{ts} = \sum_w \left(G_w \log(h_G D_w) - h_G D_w - \sum_{i=1}^{G_w} \log(i) \right). \tag{4}$$

For the pre- and post-vaccination genotype distributions, we assumed the observed total number of infections with each genotype followed a multinomial distribution with the probability p_g (where g=1 corresponds to G1, g=2 corresponds to G2,…, and g=5 corresponds to G9) given by the model-predicted genotype distribution, such that the log-likelihood of the pre-vaccination (LL_{pv}) and post-vaccination (LL_v) genotypes distributions are given by:

$$LL_{pv} = \sum_{g=1}^{5} G_{pv,g} \log(p_{pv,g}) \tag{5}$$

$$LL_v = \sum_{g=1}^{5} G_{B,g} \log(p_{v,g}) \tag{6}$$

where $G_{pv,g}$ is the sum of $G_{w,g}$ across all weeks w prior to the introduction of vaccination (September 2007-December 2005), $G_{B,g}$ is the total number of infections with each genotype g from the post-vaccination RSNB data, and

$$p_{pv,g} = \frac{\sum_{w=1}^{328} D_{w,g}}{\sum_{g=1}^{5}\sum_{w=1}^{328} D_{w,g}}, \quad p_{pv,g} = \frac{\sum_{w=419}^{720} D_{w,g}}{\sum_{g=1}^{5}\sum_{w=419}^{720} D_{w,g}}. \tag{7}$$

(Note that week w=328 corresponds to the last week of December 2005, w=419 corresponds to September 2007 when the RSNB data begins, and w=720 corresponds to the last week of June 2013.) We scaled each of these components as follows such that they contributed approximately equally to the overall log-likelihood (LL_S):

$$LL_s = LL_{ts} + LL_{pv}\frac{T}{\sum_{g=1}^{5} G_{pv,g}} + LL_v \frac{T}{\sum_{g=1}^{5} G_{B,g}} \tag{8}$$

where T=720 is the length (in weeks) of the RVGE time series from GUH.

We started by generating 100,000 parameter sets by sampling from reasonable parameter ranges for the nine parameters to be estimated using LHS (Table S1). For each parameter set, we randomly sampled from the 10 best-fit parameter sets for the non-strain-specific model and fixed the values of $\boldsymbol{\theta}_N = \{R_0, \rho_A, b_0, \phi, \omega\}$ at their corresponding estimated values (Table 1). We then simulated the strain-specific model under each of the sampled set of parameters and evaluated the log posterior probability.

Using a simplex search algorithm starting from the 10 parameter sets that yielded the highest posterior probability, we again found there were multiple parameter sets with approximately equal support (Table S2).

To determine why we could not identify a global maximum in the posterior probability surface of the strain-specific model, we calculated the conditional posterior probability profiles around the estimated parameter set $\widehat{\theta_S}$ (where $\boldsymbol{\theta_S} = \{r_1, r_2, \sigma_{HO}, \sigma_{PH}, \sigma_{HE}, \xi, s_{RV1}, s_{RV5}, h_G\}$) that yielded the lowest negative log posterior probability (up to a normalizing constant) (Fig. S4). We varied each of the parameters over a range consistent with that observed among the various estimates while holding the other parameters fixed and calculated the negative log posterior probability. We repeated this analysis using both the (default) non-stiff explicit Runge-Kutta (4,5) solver ("ode45" in MATLAB) and a stiff ODE solver ("ode23tb" in MATLAB), since there appeared to be small amounts of integration error (e.g. Fig. S4h). The problem was accentuated using the stiff ODE solver, but the general shape of the profiles remained the same (Fig. S4). Examining some of the parameter sets that yielded a poor fit to the data, we discovered that the model had a tendency to exhibit multiannual or chaotic dynamics under certain conditions, particularly following vaccine introduction. This led to multiple peaks and valleys in the posterior probability surface (e.g. Fig. S4a), such that small changes in one of the parameters could lead to large changes in the log posterior probability (e.g. Fig. S4a, Fig. S6). Some of the conditional posterior probability profiles did not appear to exhibit unique maxima (Fig. S4a-e), suggesting that the (unconditional) likelihood profiles could not be used to derive confidence intervals for the parameters.

To address the problem with the integration error, we first calculated the residuals of the conditional posterior probability profile for one of the parameters (ξ) versus the best-fit polynomial function, which appeared to provide a good approximation to the posterior probability surface (Fig. S5). We then scaled the log posterior probability such that there would be at most a 5% difference in the posterior probability at or near an optimum.

In order to fully describe the multi-modal posterior probability surface, we used importance sampling to estimate the posterior distribution of the model parameters, as described in the main text. For this analysis, we fixed $\boldsymbol{\theta_N}$ at the parameter set corresponding to the to the parameter set $\widehat{\theta_S}$ that yielded the

highest posterior probability identified from the simplex search ($\boldsymbol{\theta}_N$ corresponds to Model 1 in Table 1; $\widehat{\theta_S}$ corresponds to Model 2 in Table S2). However, as a sensitivity analysis, we repeated the procedure using the parameter set $\boldsymbol{\theta}_N$ that yielded the highest posterior probability for the non-strain-specific model fit to the Carenet-NCSF data (Model 10 in Table 1). While the posterior distribution of some of the parameters was shifted slightly higher, the qualitative conclusions remained the same (Fig. S7).

Figure S1. Sources of data on vaccination coverage. (**a**) The estimated vaccination coverage with either vaccine is plotted for the sales data for Rotarix and RotaTeq (black line) and the Inter Mutualistic Agency (IMA-AIM) data on reimbursements for both vaccines (grey lines). (**b**) Vaccine coverage with Rotarix (purple) and RotaTeq (pink). The thicker lines represent the sales data, while the thinner lines represent the IMA-AIM data. The solid lines represent coverage with at least one dose of either vaccine, while the dashed lines represent coverage with the complete course (2 doses for Rotarix, 3 doses for RotaTeq). Vaccine coverage was calculated as described in the Methods.

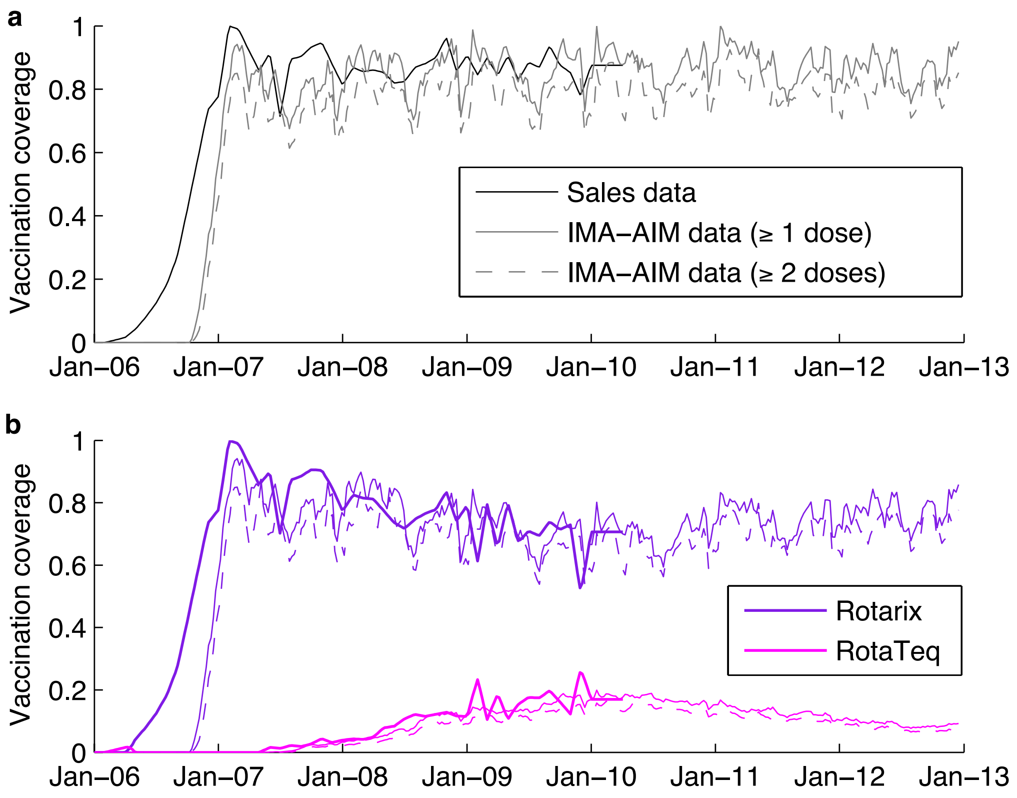

Figure S2. Correlation between estimates of the basic reproductive number and relative infectiousness of asymptomatic cases for the non-strain-specific model. Scatter plot of the estimates of R_0 and ρ_A for the 10 best-fit parameter sets for the non-strain-specific model.

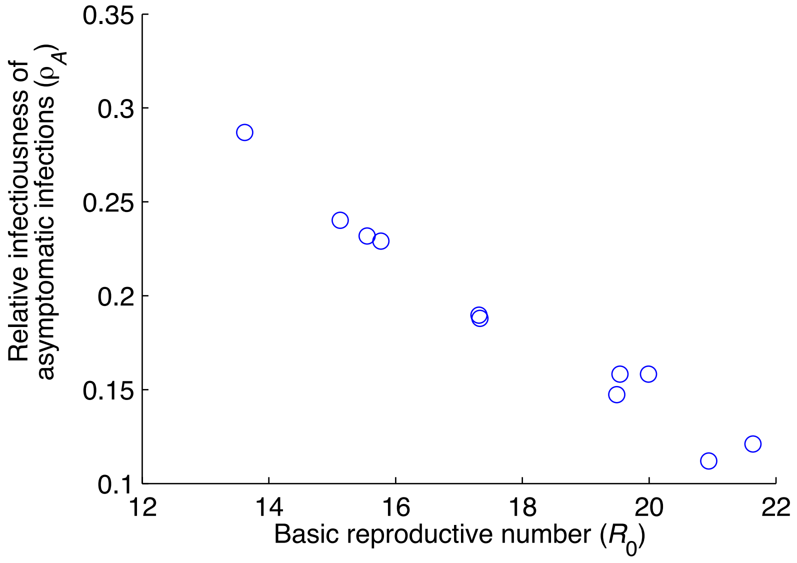

Figure S3. Top 100 parameter sets for the strain-specific model during the initial stage of model fitting. The negative log posterior probability of the model given the data is plotted against the sampled parameter value for (**a**) the relative infectiousness of the non-G1 P[8] strains compared to G1P[8] (r_1); (**b**) the relative infectiousness of G2P[4] compared to G1P[8] (r_2); (**c**) the relative risk of second infection with a partially heterotypic strain (different G-type, same P-type) (σ_{PH}); (**d**) the relative risk of second infection with a fully heterotypic strain (different G- and P-type) (σ_{HE}); (**e**) the relative risk of second infection with a homotypic strain (same G- and P-type as the strain causing first infection) (σ_{HO}); (**f**) the proportion of fully vaccinated individuals who develop a broadly heterotypic immune response (ξ); (**g**) the proportion of those vaccinated with at least one dose of RV1 who seroconvert and therefore receive any protection (s_{RV1}); (**h**) the proportion of those vaccinated with at least one dose of RV5 who seroconvert (s_{RV5}); and (**i**) the reporting fraction for severe RVGE cases presenting to GUH (h_G). The marker colour and shape corresponds to the sampled fixed parameter set from the non-strain-specific model fits.

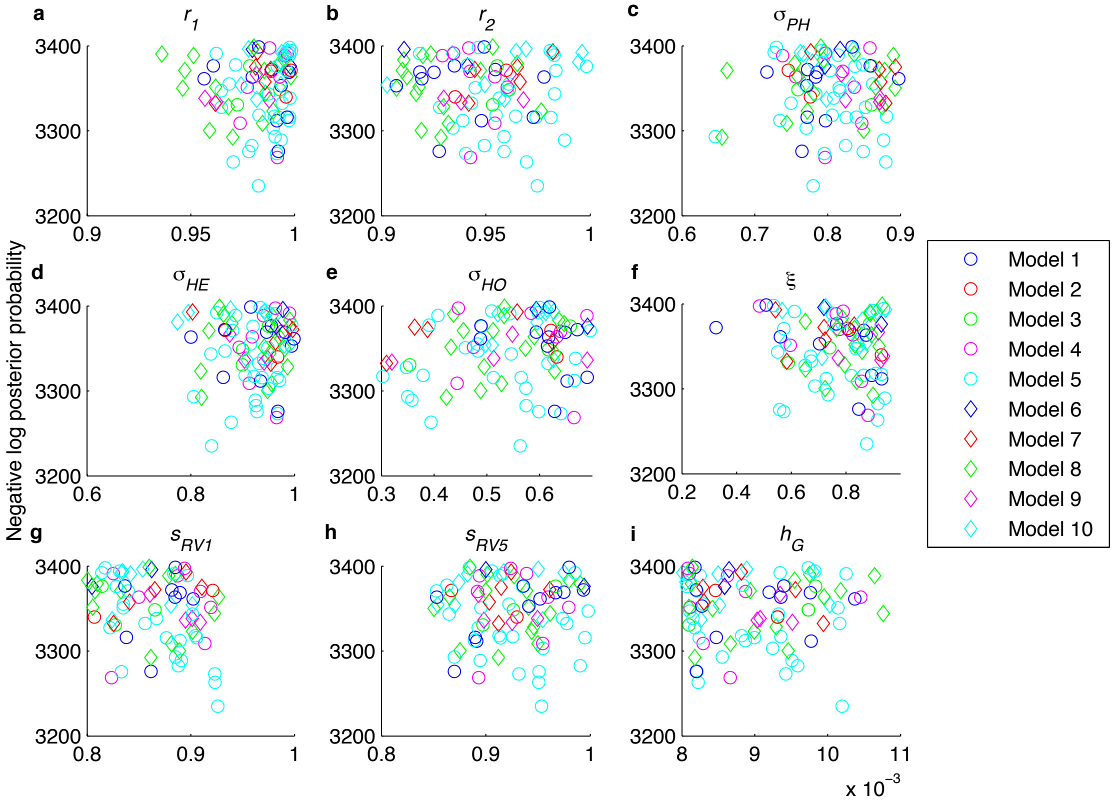

Figure S4. Conditional posterior probability profiles for the best-fit strain-specific model parameters. The negative log posterior probability of the model given the data is plotted against the sampled parameter value for (**a**) the relative infectiousness of the non-G1 P[8] strains compared to G1P[8] (r_1); (**b**) the relative infectiousness of G2P[4] compared to G1P[8] (r_2); (**c**) the relative risk of second infection with a partially heterotypic strain (different G-type, same P-type) (σ_{PH}); (**d**) the relative risk of second infection with a fully heterotypic strain (different G- and P-type) (σ_{HE}); (**e**) the relative risk of second infection with a homotypic strain (same G- and P-type as the strain causing first infection) (σ_{HO}); (**f**) the proportion of fully vaccinated individuals who develop a broadly heterotypic immune response (ξ); (**g**) the proportion of those vaccinated with at least one dose of RV1 who seroconvert and therefore receive any protection (s_{RV1}); (**h**) the proportion of those vaccinated with at least one dose of RV5 who seroconvert (s_{RV5}); and (**i**) the reporting fraction for severe RVGE cases presenting to GUH (h_G). The red lines represent the results when the model was simulated using a stiff ODE solver, while the blue lines represent the results for a non-stiff ODE solver. Only the results for the non-stiff solver are shown for (**f-i**) for clarity.

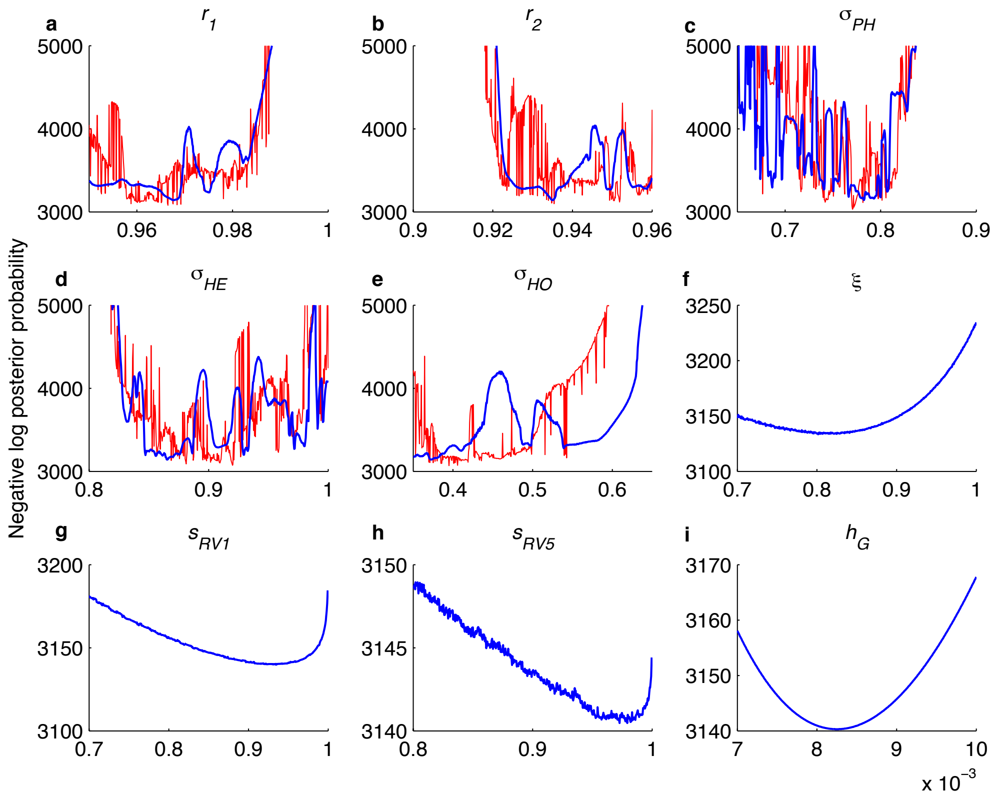

Figure S5. Analysis of error in ODE solver. (**a**) The negative log posterior probability of the model given the data for different values of ξ near the best-fit parameter set, $\widehat{\theta_S}$ is plotted in blue, while a best-fit 7th degree polynomial relationship is plotted in green. (**b**) The residuals of the model log-posterior versus the fitted relationship are plotted in green.

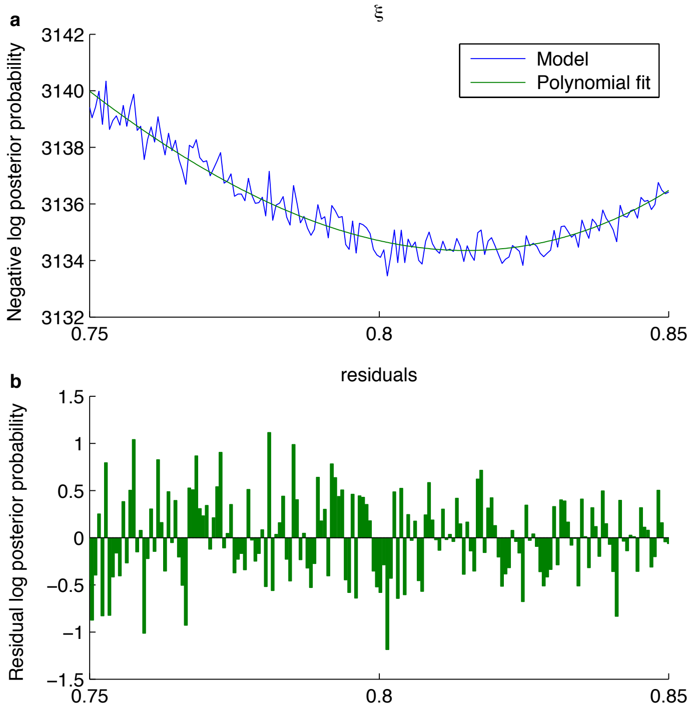

Figure S6. Example of multiannual post-vaccination dynamics. (**a**) Model-predicted number of rotavirus hospitalizations at GUH in Leuven for an example parameter set with r_1=0.9707 and all other parameter set to the best-fit estimates (solid line) and the best-fit parameter set, for which r_1=0.9676 (dashed line). (**b**) Model-predicted annual genotype distribution for the example parameter set. (**c**) Model-predicted pre-vaccination genotype distribution for the example parameter set (left) and the best-fit model (right). (**d**) Model-predicted post-vaccination genotype distribution for the example parameter set (left) and the best-fit model (right).

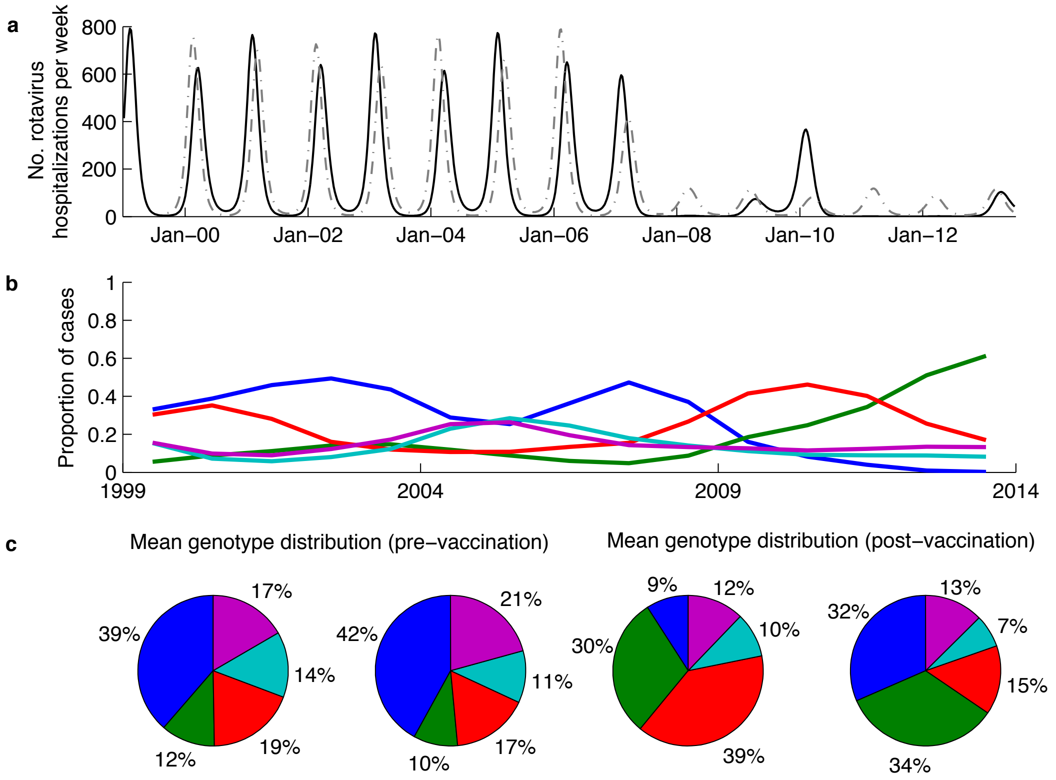

Figure S7. Sensitivity of parameter estimates for the strain-specific model to the choice of parameters estimated from the non-strain-specific model. Box plots of the posterior distributions for (**a**) the relative risk of second infection with a homotypic strain (σ_{HO}, blue), partially heterotypic strain (σ_{PH}, red), or fully heterotypic strain (σ_{HE}, green) compared to the risk of first infection; (**b**) the relative infectiousness of non-G1 P[8] strains (r_1, red) and G2 strains (r_2, green) compared to G1 strains; (**c**) proportion of vaccinees who seroconvert and thus receive any benefit of vaccination with RV1 (s_{RV1}, purple) or RV5 (s_{RV5}, pink), and the proportion of vaccinees who receive broadly heterotypic protection equivalent to two natural infections (ξ, grey); and (**d**) the reporting fraction (h_G, black) for moderate-to-severe RVGE cases in Belgium to be hospitalized and G-typed at GUH. The coloured boxes represent the posterior distribution for the $\boldsymbol{\theta}_N$ corresponding to the best-fit parameter set for the strain-specific model, while the white boxes represent the posterior distribution for the $\boldsymbol{\theta}_N$ corresponding to the parameter set the yielded the second highest log-likelihood during the simplex search.

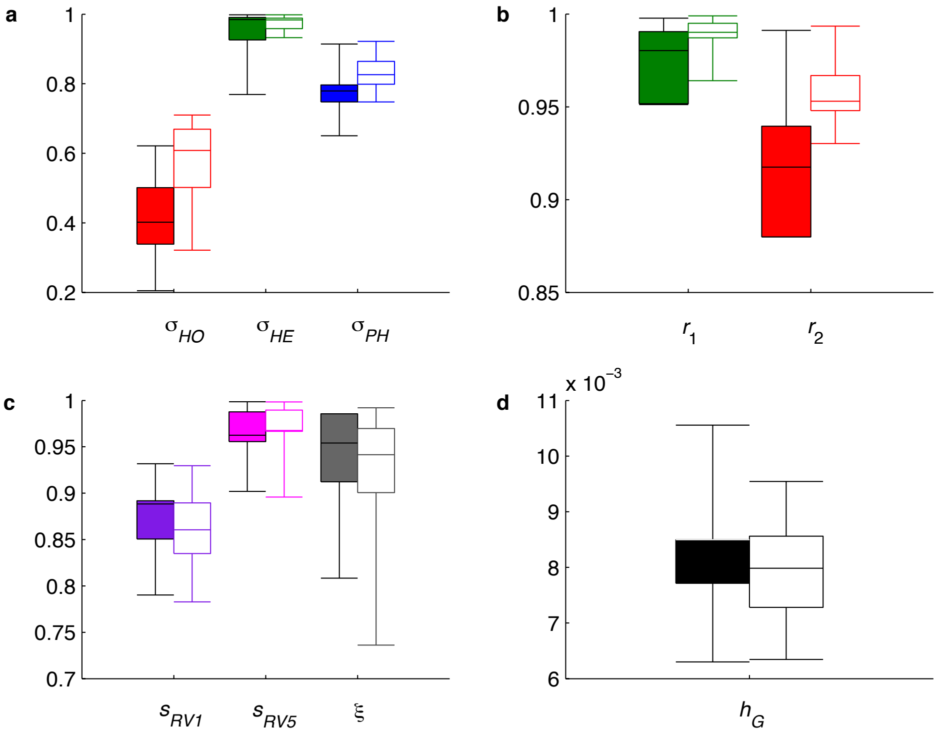

Table S1. Prior distributions for the parameters of the strain-specific model.

Parameter	Symbol	Initial range	Prior distribution
Relative infectiousness of non-G1 P[8] strains	r_1	0.9 to 1	Uniform(0,1)
Relative infectiousness of G2P[4] strains	r_2	0.9 to 1	Uniform(0,1)
Relative risk of second infection with a partially heterotypic strain	σ_{PH}	0.6 to 0.9	Uniform(0,1)
Relative risk of second infection with a completely heterotypic strain	σ_{HE}	0.6 to 1	Uniform(0,1)
Relative risk of second infection with a homotypic strain	σ_{HO}	0.3 to 0.7	Uniform(0,1)
Proportion of vaccinees receiving protection equal to two natural infections	ξ	0.2 to 0.9	Uniform(0,1)
Proportion of vaccines who seroconvert to RV1 vaccine	s_{RV1}	0.8 to 1	Beta(76,12.4)
Proportion of vaccines who seroconvert to RV5 vaccine	s_{RV5}	0.8 to 1	Beta(42,2.2)
Hospitalization/reporting rate for GUH, Leuven	h_G	0.008 to 0.011	Uniform(0,1)

Table S2. Parameter estimates from the simplex search for the strain-specific model fit to the GUH data.

**Parameter**	**Sym-bol**	**Model 1**	**Model 2**	**Model 3**	**Model 4**	**Model 5**	**Model 6**	**Model 7**	**Model 8**	**Model 9**	**Model 10**
Relative infectiousness of non-G1 P[8] strains	r_1	0.956	0.968	0.991	0.992	0.993	0.992	0.990	0.990	0.995	0.991
Relative infectiousness of G2P[4] strains	r_2	0.926	0.935	0.934	0.951	0.916	0.941	0.911	0.922	0.914	0.945
Relative risk of second infection with a partially heterotypic strain	σ_{PH}	0.809	0.782	0.733	0.695	0.711	0.726	0.732	0.729	0.666	0.729
Relative risk of second infection with a completely heterotypic strain	σ_{HE}	0.890	0.865	0.973	0.866	1.000	0.936	0.964	0.930	0.963	0.920
Relative risk of second infection with a homotypic strain	σ_{HO}	0.360	0.373	0.588	0.543	0.590	0.592	0.619	0.614	0.539	0.589
Proportion of vaccinees receiving protection equal to two natural infections	ξ	0.726	0.876	0.889	0.829	0.855	0.796	0.886	0.908	0.885	0.754
Proportion of vaccines who seroconvert to RV1 vaccine	s_{RV1}	0.854	0.905	0.847	0.829	0.727	0.830	0.789	0.868	0.847	0.764
Proportion of vaccines who seroconvert to RV5 vaccine	s_{RV5}	0.937	0.957	0.944	0.936	0.921	0.851	0.930	0.947	0.983	0.917
Hospitalization/reporting rate for GUH, Leuven	h_G	0.0091	0.0081	0.0083	0.0080	0.0084	0.0081	0.0081	0.0084	0.0082	0.0078
Log posterior		3,181	3,141	3,150	3,198	3,166	3,162	3,165	3,166	3,156	3,171
